# A trait-based framework for seagrass ecology: Trends and prospects

**DOI:** 10.3389/fpls.2023.1088643

**Published:** 2023-03-20

**Authors:** Agustín Moreira-Saporiti, Mirta Teichberg, Eric Garnier, J. Hans C. Cornelissen, Teresa Alcoverro, Mats Björk, Christoffer Boström, Emanuela Dattolo, Johan S. Eklöf, Harald Hasler-Sheetal, Nuria Marbà, Lázaro Marín-Guirao, Lukas Meysick, Irene Olivé, Thorsten B. H. Reusch, Miriam Ruocco, João Silva, Ana I. Sousa, Gabriele Procaccini, Rui Santos

**Affiliations:** ^1^ Faculty for Biology and Chemistry, University of Bremen, Bremen, Germany; ^2^ Algae and Seagrass Ecology Group, Department of Ecology, Leibniz Centre for Tropical Marine Research, Bremen, Germany; ^3^ CEFE, Univ Montpellier, CNRS, EPHE, IRD, Montpellier, France; ^4^ Systems Ecology, A-LIFE, Vrije Universiteit Amsterdam, Amsterdam, Netherlands; ^5^ Centre d’Estudis Avançats de Blanes, Blanes, Spain; ^6^ Department of Ecology, Environment and Plant Sciences (DEEP), Stockholm University, Stockholm, Sweden; ^7^ Åbo Akademi University, Environmental and Marine Biology, Åbo, Finland; ^8^ Department of Integrative Marine Ecology, Stazione Zoologica Anton Dohrn, Naples, Italy; ^9^ University of Southern Denmark, Odense, Denmark; ^10^ Global Change Research Group, Institut Mediterrani d’Estudis Avançats (IMEDEA, CSIC-UIB), Esporles Illes Balears, Spain; ^11^ Oceanographic Center of Murcia, Spanish Institute of Oceanography (IEO-CSIC), Murcia, Spain; ^12^ Helmholtz Institute for Functional Marine Biodiversity (HIFMB) at the University of Oldenburg, Oldenburg, Germany; ^13^ Alfred Wegener Institute Helmholtz Centre for Polar and Marine Research, Bremerhaven, Germany; ^14^ School of Geographical and Earth Sciences, University of Glasgow, Glasgow, United Kingdom; ^15^ Marine Evolutionary Ecology, Division of Marine Ecology, GEOMAR Helmholtz Center for Ocean Research Kiel, Kiel, Germany; ^16^ Centro de Ciências do Mar, Universidade do Algarve, Campus de Gambelas, Faro, Portugal; ^17^ CESAM – Centre for Environmental and Marine Studies, Department of Biology, University of Aveiro, Campus Universitário de Santiago, Aveiro, Portugal

**Keywords:** functional ecology, trait-based approach, seagrass traits database, ecosystem service vulnerability, response-effect framework

## Abstract

In the last three decades, quantitative approaches that rely on organism traits instead of taxonomy have advanced different fields of ecological research through establishing the mechanistic links between environmental drivers, functional traits, and ecosystem functions. A research subfield where trait-based approaches have been frequently used but poorly synthesized is the ecology of seagrasses; marine angiosperms that colonized the ocean 100M YA and today make up productive yet threatened coastal ecosystems globally. Here, we compiled a comprehensive trait-based response-effect framework (TBF) which builds on previous concepts and ideas, including the use of traits for the study of community assembly processes, from dispersal and response to abiotic and biotic factors, to ecosystem function and service provision. We then apply this framework to the global seagrass literature, using a systematic review to identify the strengths, gaps, and opportunities of the field. Seagrass trait research has mostly focused on the effect of environmental drivers on traits, i.e., “environmental filtering” (72%), whereas links between traits and functions are less common (26.9%). Despite the richness of trait-based data available, concepts related to TBFs are rare in the seagrass literature (15% of studies), including the relative importance of neutral and niche assembly processes, or the influence of trait dominance or complementarity in ecosystem function provision. These knowledge gaps indicate ample potential for further research, highlighting the need to understand the links between the unique traits of seagrasses and the ecosystem services they provide.

## 1 Introduction

Trait-based response-effect frameworks (hereafter TBFs) have been extensively used in terrestrial plant ecology (Suding et al., 2008; Díaz et al., 2013). TBFs are based on the study of traits, which capture the form and function of organisms, and are defined as “any morphological, physiological or phenological heritable feature measurable at the individual level, from the cell to the whole organism, without reference to the environment or any other level of organization” (Violle et al., 2007 as modified by Garnier et al., 2016). Traits are categorized into response and effect traits. Hence, the structure of a plant community is the result of the environmental filters and biotic interactions that exclude phenotypes that do not possess appropriate response trait values (Weiher and Keddy, 1995; Díaz et al., 1998; Belyea and Lancaster, 1999). Effect traits, on the other hand, influence how the organism affects ecosystem functions and they are therefore controlled by the distribution of trait values shaping the community (Garnier et al., 2016).

**Figure 1:**
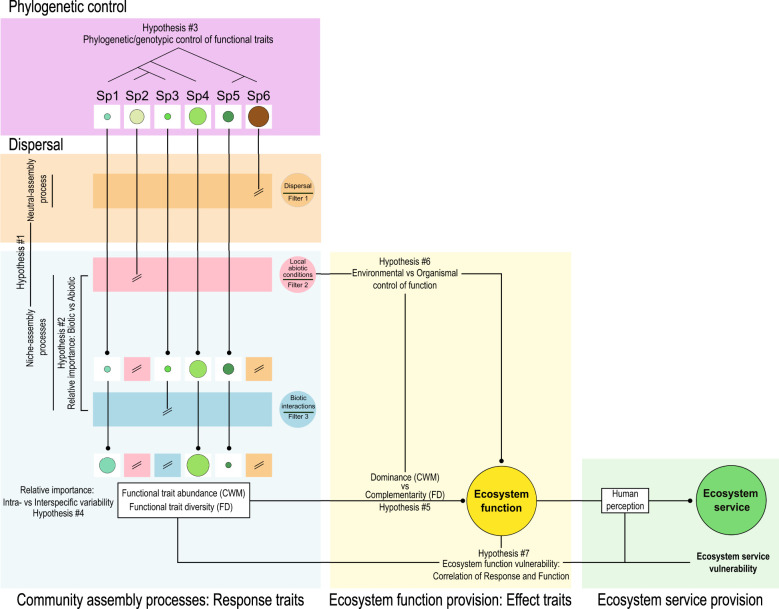
Conceptual TBF (trait-based response-effect framework) synthetized combining the concepts introduced by Lavorel and Garnier (2002); Suding et al. (2008); Violle et al. (2012); Díaz et al. (2013) and Mouillot et al. (2013). A given array of species at the regional level (Sp1-Sp6) are influenced by several filters until the final local community assemblage. The circles indicate the species abundance at the regional level (top) and at the local level after the filters (bottom). Traits can be used to study if community assemblage is a neutral or niche assembly process, meaning that it is mainly stochastic or affected by environmental drivers (Filters 1 and 2, Hypothesis #1). Once settled, the relative importance of the abiotic filter (i.e. environmental drivers) and the biotic filter (Filter 3) (i.e. competition for resources and biotic interactions) can be studied through trait convergence and divergence (Hypothesis #2). Functional trait abundance (CWM) and diversity (FD) can be calculated in a community to study the influence of traits in ecosystem function delivery (Hypothesis #5). Functional traits can, however, be phylogenetically controlled (Hypothesis #3) and their inter- and intraspecific variability may change among species and communities (Hypothesis #4). Ecosystem function delivery not only depends on traits, but also the environmental constraints may play a central role in it (Hypothesis #6). The correlation of the ecosystem function delivery and response will determine the function vulnerability (Hypothesis #7). Finally, ecosystem functions and their perception by humankind determine the ecosystem services provided and, therefore, their vulnerability.

There are many examples of the use of TBFs in terrestrial plant ecology. Functional trait diversity explains more variation of community biomass than species richness (Roscher et al., 2012); community‐weighted means of leaf dry matter content can be used to explain variations in digestibility, which is a critical component of herbage nutritive value, a major service delivered by grasslands (Gardarin et al., 2014); litter decomposition is not only controlled by the abiotic environment, but mostly by species-level plant traits (Cornwell et al., 2008; Tardif et al., 2014). The general relevance of the TBF to the study of terrestrial plant ecology has triggered its development in marine ecology (e.g. Solan et al., 2004; Follows et al., 2007; Andersen and Pedersen, 2009; Edwards et al., 2013; Elleouet et al., 2014). TBFs enable generalized predictions of community composition and function of any type of ecosystem across organizational and spatial scales, independent of taxonomy (Shipley et al., 2016), which allows for the testing of a variety of ecological hypotheses. To illustrate the concepts that have been developed in trait-based research, a conceptual TBF has been compiled (**Figure 1**), based on the seminal works by Lavorel and Garnier (2002) and Suding et al. (2008), which also considers phylogeny (Díaz et al., 2013) and intraspecific variability (Violle et al., 2012) using modern analytical methods (Mouillot et al., 2013).

First, community assembly processes encompass the mechanisms underlying the composition and structure of communities in response to environmental variation (McGill et al., 2006; Weiher et al., 2011; Grime and Pierce, 2012), from dispersal to the influence of abiotic and biotic factors. For plants, dispersal into a local community (**Figure 1**: Filter 1) is partly controlled by stochastic processes occurring at a geographical scale and random local events, which drive colonization and local extinctions respectively and are poorly related to the traits of organisms (Weiher et al., 2011). The abiotic filter (**Figure 1**: Filter 2) determines which species can establish due to the influence of local environmental conditions, the availability of resources, and the disturbance regime (Wilson, 2011). This defines the fundamental niche of the species. The biotic filter (**Figure 1**: Filter 3) corresponds to the positive and negative interactions between living organisms within communities and determines the set of coexisting neighboring species (Tilman, 1985). It is the realized niche of the species along the range of possibilities from competitive exclusion (Gause, 1937) to facilitation (Maxwell et al., 2017). These niche assembly processes define how local communities assemble from the regional species pool through the filtering of abiotic and biotic factors (Keddy, 1992) that, together with stochastic processes, explain the characteristics of local communities (Vellend, 2010).

To understand which metrics might be useful for detecting which assembly process predominates in shaping a community, it is helpful to envisage species trait values as coordinates (e.g. along axes of variation in multivariate analysis such as principal component analysis) locating species in the functional space (see Mouillot et al., 2013). Studying whether the functional coordinates of a species are sorted out from the local pool is random or the consequence of their response to the environmental drivers provides the grounds to test the niche and neutral assembly theories (Garnier et al., 2016) (**Figure 1**: Hypothesis #1).

The functional trait structure of the local community can be convergent (showing high similarity among functional traits in co-existing species) or divergent (showing dissimilarity among functional traits in co-existing species) (Grime, 2006; Cornwell and Ackerly, 2009; Bernard-Verdier et al., 2012; Gross et al., 2013) depending on the relative importance of the abiotic and biotic filtering on the community (**Figure 1**: Hypothesis #2). Abiotic factors tend to dominate the trait distributions when they set major physico-chemical constraints on the ecosystem, which then leads to a convergent distribution, whereas biotic factors dominate when there are few or weak abiotic constraints and there is room for increasing competition (Weiher et al., 1998; Grime, 2006), which tends to lead to competitive exclusion and thereby a divergent distribution (but see discussion in Mayfield and Levine, 2010).

Functional traits can be phylogenetically conserved or they can diverge strongly at the tips of the phylogeny, thereby reflecting relatively recent evolutionary trait change (**Figure 1**: Hypothesis #3). Therefore, the measurement of phylogenetic diversity (PD) can be an indicator of functional trait diversity (FD) (Forest et al., 2007), even though there is a considerable debate on this topic (Garnier et al., 2016). Indeed, the correlation between PD and FD is not universal, and high PD can generate many assemblages that have a lower FD than randomly chosen sets of species (Mazel et al., 2018).

Intraspecific trait variability can constitute a relatively large part of overall community-level trait variability (Violle et al., 2012). Therefore, it is fundamental to determine the relative importance of inter- vs intraspecific variability (**Figure 1**: Hypothesis #4). Violle et al. (2012) showed the importance of including intraspecific variability to get a better understanding of the environmental filters acting on the vegetated community rather than using mean trait values per species present in the community. This was a revision of the concepts of alpha and beta niches (Pickett and Bazzaz, 1978), which allow understanding the effects of environmental filters on intraspecific and interspecific trait variability (Ackerly and Cornwell, 2007).

Effect traits allow to scale up from the functioning of an individual to that of ecosystems (Grime, 1998; Chapin et al., 2000; Lavorel and Garnier, 2002; Díaz et al., 2007). Two different and non-exclusive hypotheses have been formulated to relate the functional structure of communities to ecosystem properties: dominance (mass-ratio effect) and niche complementarity. The dominance hypothesis stipulates that the functional traits of the dominant species will be the predominant influence on the ecosystem function (Grime, 1998; Smith and Knapp, 2003), this being proportional to its abundance in the community (Garnier et al., 2004; Díaz et al., 2007; Violle et al., 2007). The metric used to test this hypothesis is the community weighted mean (CWM). By contrast, the niche complementarity hypothesis stipulates that the presence of functionally different species, which use environmental resources in a complementary manner, will positively influence ecosystem functioning (Loreau and Hector, 2001; Tilman, 2001; Eviner and Chapin, 2003; Díaz et al., 2006; Petchey and Gaston, 2006). It is therefore hypothesized that positive relations exist between ecosystem functions and functional diversity (FD). These two hypotheses are not mutually exclusive, and it is possible that both are important in influencing ecosystem functions (Díaz et al., 2007, **Figure 1**: Hypothesis #5). More evidence has been found, however, for a relation between dominance and function (Garnier et al., 2016). A drawback in the study of function provision is that some functions may not be correlated with traits under constraining environmental factors, not allowing for the determination of causality between trait and function. Environmental factors should be, therefore, controlled for in a “common garden” or statistically with structured equation models (Grace et al., 2007; Shipley, 2010) (**Figure 1**: Hypothesis #6) to disentangle the links between environment, trait and function.

Ecosystem services are defined as the capacity of natural processes and components to provide goods and services that satisfy human needs, directly or indirectly (de Groot et al., 2002). The definition of an ecosystem service is contingent upon human perception and needs, and therefore each ecosystem service has underlying functions that are biologically measurable. The importance of the concept of ecosystem service is the possibility to integrate ecosystem functions in management and policy. Díaz et al. (2013) introduced the concept of ecosystem service vulnerability, based on the idea that the security of ecosystem functions depends on how the effects and tolerances of organisms (which both depend on combinations of functional traits) correlate across species. Therefore, the correlation of the response and effect traits of organisms can determine the vulnerability of an ecosystem function (**Figure 1**: Hypothesis #7). The final step in the TBF proposed above is the translation of effect traits from ecosystem functions to ecosystem services. Effect traits driving ecosystem service provision are, therefore, a tool to understand the link between organism, function and service, and the vulnerability of the service provision under a changing environment.

Despite the wide application of TBFs in terrestrial plant ecology, its application has been very scarce in seagrasses. Seagrasses are a polyphyletic group of basal monocotyledonous angiosperms belonging to four families in the Alismatales: Posidoniaceae, Zosteraceae, Cymodoceaceae and Hydrocharitaceae. Limited to coastal areas, they occupy a global surface of about 160 387 km^2^ (<0.2% of the ocean’s surface, McKenzie et al., 2020). The colonization of marine habitats from terrestrial wetland habitats occurred exclusively from this monocotyledonous order and took place in four independent and parallel evolutionary events (Les et al., 1997; Janssen and Bremer, 2004; Waycott et al., 2006). From an evolutionary timescale perspective, this colonization was contingent upon a number of critical adaptations, which partially reverted many of the original key adaptations of flowering plants to terrestrial life. These adaptations are reflected in specific genomic losses and gains (Golicz et al., 2015; Lee et al., 2016; Olsen et al., 2016; Lee et al., 2018), with adaptive changes in sets of genes associated with central biological pathways (Wissler et al., 2011). Despite their successful adaptation to the marine realm and wide distribution in most coastal areas around the world, seagrasses exhibit very low species richness (60-70 species) compared to other groups in the Alismatales, which is possibly partially compensated by pronounced local adaptation (or intraspecific variability) within species (e.g. Jueterbock et al., 2016; Dattolo et al., 2017; Jahnke et al., 2019).

All seagrass species share a similar morphology with basal meristems that form strap-like leaves grouped in shoots connected by rooted rhizomes in the sediment. Their low morphological diversity is possibly the result of a convergent evolution to the submerged lifestyle in a hydrodynamically active and saline environment (Arber, 1920; Les et al., 1997). Unfortunately, the coastal habitat colonized by seagrasses is under high and increasing anthropogenic pressure. Consequently, seagrasses are under decline worldwide due to multiple local (Burkholder et al., 2007; Unsworth et al., 2018; Moreira-Saporiti et al., 2021a) and global pressures (Orth et al., 2006; Waycott et al., 2009; Turschwell et al., 2021). Reversal of this negative trend, however, is possible (Lefcheck et al., 2018; de los Santos et al., 2019; Sousa et al., 2019; Dunic et al., 2021; Turschwell et al., 2021) when appropriate management and conservation actions are implemented.

Much seagrass research to date has measured responses in various plant traits to environmental variation, to i) better understand seagrass biology and ecology (Sousa et al., 2017), ii) prevent their decline (Fernandes et al., 2019), iii) restore degraded ecosystems (Paulo et al., 2019; Lange et al., 2022), or iv) predict their fate under future global change scenarios (Hyndes et al., 2016). Synthesis of the existing data on seagrass response to the environment has been used to identify potential indicators for assessing the health of seagrass ecosystems (Roca et al., 2016). Additionally, there is a good understanding that the sole presence of seagrass is enough for the provisioning of functions like invertebrate habitat (Virnstein et al., 1983) or the modification of the inorganic carbon system (Unsworth et al., 2012). The provisioning of these functions, however, must be underpinned by the traits of the component species or genotypes, but the link between seagrass traits and functions has been resolved in only a handful of examples (e.g. Fonseca and Callahan, 1992; Hendriks et al., 2008; Gustafsson and Boström, 2011; Hendriks et al., 2014). At present, we lack a comprehensive picture and predictive framework of how key seagrass traits underpin the resistance and resilience of seagrass species to current and future pressures, and their relation to ecosystem functions and services.

In order to push seagrass research forward, we compiled the existing knowledge on seagrass trait research and pointed its knowledge gaps and research possibilities. We carried out a systematic review of the seagrass literature with the goal of quantifying the use of TBFs in the assessment of seagrass responses, ecosystem functions and services and to identify the gaps of knowledge in this field, including (1) how frequently trait-based research has been adopted in seagrass ecological research and how many of these studies could be classified as TBFs (as defined by the seminal work from Lavorel and Garnier, 2002), (2) which of the methodologies, hypotheses and theories introduced by TBFs have been already studied in seagrass communities in relation to their traits and (3) identify under- and over-studied traits, drivers and functions in seagrass research, with examples from the literature. The conceptualization of the results of the literature review under a TBF will allow the exploration of the research gaps and indicate future research pathways in seagrass ecology, specifically focusing on the ecosystem function and service provision and vulnerability.

## 2 Methods

We followed the ROSES protocol (Haddaway et al., 2018) for a literature review (metadata of the review can be found in the Supplementary Material 1). We identified 21,100 publications of potential relevance within the Google Scholar database using the query “Seagrass trait” and “*Seagrass species* trait” (“*Seagrass species”* being the currently accepted names of all seagrass species). To guarantee that the focus of the publication was on the study of trait-based research, the word trait had to be present in the title, abstract and/or keywords of the publication, elsewise the publication was not included in the review process. We acknowledge that this search query would leave out literature studying seagrass traits, but not using the terminology “trait”. However, this was the only way to ensure the focus of the review in the study of seagrass traits and trait-based research. The number of publications was limited to those in English. The temporal range of the sample was restricted to the limitations of the database itself, i.e. publications included the range from 1988 through March 2022. Using the above screening criteria, the initial number of publications was reduced to 380. From these 380 publications, 137 were discarded as they referred to the study of seagrass-associated fauna, benthic macroalgae within seagrass meadows and seagrass epiphytes; 12 duplicates and 19 misclassified publications were also discarded. 19 more publications were discarded as they were gray literature. The final database was sized down to 193 relevant publications. The complete database with the categorization of the publications can be found in the **Supplementary Material 2**.

For goal (1), we counted the number of studies including the word “trait” and the number of studies in which an existing TBF (as defined by Lavorel and Garnier, 2002) was used to test a hypothesis or research question. For goal (2), we categorized the studies in *a priori* categories derived from the TBF presented above (**Figure 1**). Lastly, for goal (3), we created an *a priori* classification of seagrass traits and *a posteriori* classification with the environmental drivers and ecosystems functions found in the literature. We made the final figures using the software R with the package ggplot (Wickham, 2016; R Core Team, 2022) and InkScape (v 0.92).

## 3 Results and discussion

### 3.1 Seagrass TBF studies and studies including the word trait

**Figure 2:**
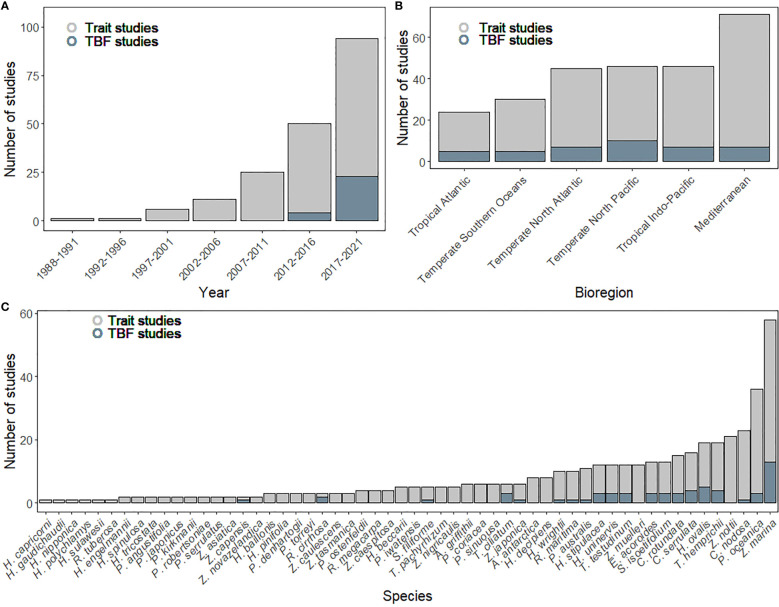
Number of studies included in the systematic review classified by **(A)** Year, **(B)** Bioregion and **(C)** Seagrass species. The light gray color indicates the number of studies including the word “trait” and the dark gray color the number of studies classified as using a trait-based frameworks (TBF). Studies from the year 2022 (n=5 until March 31st) are not included in this figure.

Trait-based response-effect frameworks, TBFs, are currently underexplored in seagrass research. The number of studies including the word “trait” increased steadily since the first study from the year 1988, reaching a maximum in the last five years (n=73, **Figure 2A**). Only 29 studies were found to use existing TBFs, accounting for only 15% of the total. The “trait-based approach” was first developed in 2002 (Lavorel and Garnier, 2002) for terrestrial plants, and it does not appear in the seagrass literature until 2012. This indicates that the body of knowledge available from terrestrial plant ecology has been under-utilized by seagrass researchers.

Studies focusing on seagrass traits have been mainly developed in the Mediterranean bioregion (27%, **Figure 2B**), while the Tropical Atlantic (9%) and Temperate Southern Oceans (11%), showed the lowest number of studies. TBF studies have been homogeneously performed in all bioregions, with the Temperate North Pacific showing the highest number (24%). Differences across bioregions could be attributed to the differential use of the term “trait” across research groups and the seagrass species that are the focus of their study. As a consequence, while the widespread species *Zostera marina* accounted for 30% of trait studies, *Posidonia oceanica*, endemic to the Mediterranean Sea, accounted for 19% (**Figure 2C**). This result indicates a certain bias in the use of the trait nomenclature in certain species like *P. oceanica*, while it simultaneously highlights the problem of research bias and inference from unique species to the others, specifically when trait responses (Viana et al., 2020) can be species-specific.

### 3.2 Dispersal and settlement in seagrass communities: Challenging the neutral assembly theory

**Figure 3:**
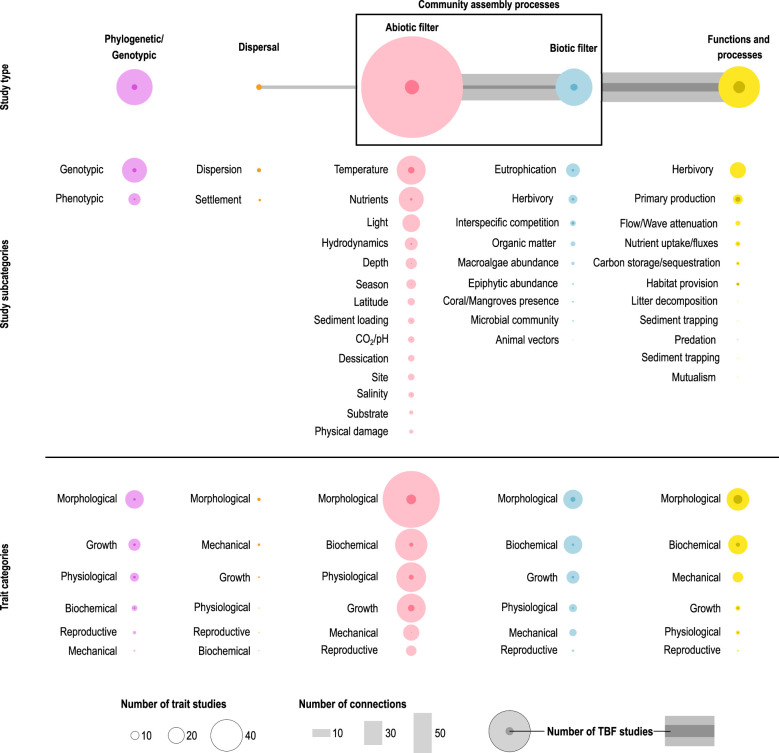
Results of the systematic literature review adapted to the conceptual TBF proposed by this manuscript. The studies were classified in “Study types” (top), “Study subcategories” (middle, *a posteriori* classification with the environmental drivers and ecosystem functions found in the review process) and “Trait categories” (bottom, trait classification decided before the review process). The size of the circle indicates the number of trait studies. The darker circles within indicate the number of studies classified as using TBFs. The horizontal lines indicate the number of connections among the “Study types”. The darker lines indicate the number of connections among study types using a TBF.

We found seven studies that linked seagrass dispersal and settlement to traits (3.6% of the total, **Figure 3**), suggesting that seagrass dispersal can be predicted by the traits. Target traits can change according to the life stage of the seagrass plant (seed or vegetative fragment) (Orth et al., 2007; McMahon et al., 2014; Bryan-Brown et al., 2017). In the case of seeds, traits such as flotation capacity and digestibility determine the distance they can disperse and the effectiveness of using animal vectors, respectively (Wu et al., 2016). Seeds also show a high degree of intraspecific variability in size, which determines their settling velocity and dispersal potential (Delefosse et al., 2016). When it comes to settlement, traits like germination rate can be site specific and negatively affected by increasing temperature in *Z. marina* (Cabaço and Santos, 2010). In addition, the current velocity in the settlement area and the stiffness and flexibility of surrounding shoots limit the settlement capacity of seagrass seeds (Bouma et al., 2009). In the case of vegetative fragments, the plant morphology can partially control their dislodgement resistance, whereas the age and rooting rate determine their capacity for settlement (Lai et al., 2018). Vegetative fragments have the potential for long distance dispersal thanks to long lasting shoot buoyancy and survival, allowing the colonization of new areas (Berković et al., 2014). Additionally, fruits of certain seagrass species allow for long distance dispersal as well, as it is the case of *Posidonia australis* (10s to 100s of kilometers, Ruiz-Montoya et al., 2015).

The identification of traits controlling dispersal and settlement of seeds and vegetative fragments challenges the perception of dispersal as a stochastic and unpredictable process. In addition, abiotic (temperature, current velocity, wave disturbance and exposure) and biotic (animal vectors, shoot stiffness) factors as well as a combination of these exemplified by the seascape mosaic formed by the plants on an unvegetated substrate affect their dispersal and settlement success. The neutral assembly process hypothesis (**Figure 1**: Hypothesis #1), while not formally tested, seems to be false in the case of seagrasses (**Table 1**). However, only a small number of studies investigated this question. Further research is needed to test this hypothesis at different scales, with null models as a fundamental tool to assess the relative importance of purely stochastic and niche assembly processes (Mori et al., 2015). This hypothesis has been tested in other marine organisms, including fish communities (Ford and Roberts, 2018), which assemble neutrally at the regional scale but not at the local scale, and woody plants, whose dispersal and settlement are primarily trait-driven (Duarte et al., 2010).

**Table 1:** Studies included in the literature review that linked dispersal and settlement with seagrass traits.

Hypothesis #1 Neutral assembly process: Preliminary rejected					
Process	Dispersal unit	Relevant traits	Abiotic filter	Biotic filter	Sources
Dispersal	Seed	Sinking rate, seed size	Current velocity	Animal vectors	Berković et al. (2014); Delefosse et al. (2016); Wu et al. (2016); Lai et al. (2018)
	Vegetative fragment	Shoot buoyancy, shoot viability, fragment breakage, shoot growth rate, spathe release rate, dislodgement resistance	Current velocity, burial conditions	–	
Settlement	Seed	Seed weight, germination rate	Temperature, current velocity, scouring	Shoot size and stiffness of surrounding seagrass	Bouma et al. (2009); Cabaço and Santos (2010); Lai et al. (2018)
	Vegetative fragment	Fragment age, rooting rate	Current velocity	–	

### 3.3 Community assembly processes: The abiotic and biotic filters in seagrass communities

Much research has been performed on the responses of seagrass traits to environmental drivers, making a total of 72% of the studies found in the literature review. There was, however, a large imbalance between the study of seagrass traits under the abiotic (89%) vs the biotic (11%) filter (**Figure 3**). This indicates that the knowledge of seagrasses is focused on the study of its fundamental niche, i.e. the major physico-chemical constraints in the system. For example, the vertical zonation of tropical seagrasses was explained by physiological traits controlling their ability to tolerate high irradiances and nutrient inputs (Björk et al., 1999). Other examples focus on the assessment of the fundamental niche of individual species. *Halophila decipiens* occupies a wide range of irradiances and temperatures, due to its phenotypic plasticity (Gorman et al., 2016). *Z. marina* has a low niche specialization in the Baltic Sea, allowing this species to exist under variable environmental conditions in comparison to other macrophytes (Herkül et al., 2018). The three most frequent abiotic drivers studied were temperature (28.3%), nutrients (24.4%) and light (17.3%), whereas the least studied include the effect of freshwater input (1.5%) or metal pollution (2.4%).

Traits have been therefore used as indicators of environmental change, and their response is both driver and species-specific. This can be illustrated using the example of temperature. An increase in temperature within the optimal range fosters leaf growth (*Thalassia hemprichii*: Viana et al., 2020; *Enhalus acoroides*: Artika et al., 2020) and leaf size (*Z. marina*: Young Kim and Seob Choi, 2004; Ondiviela Eizaguirre et al., 2018; DuBois et al., 2019; *Halodule wrightii*: Sordo et al., 2011; *Zostera noltei*: Ondiviela Eizaguirre et al., 2018; *T. hemprichii*: Viana et al., 2020; *Cymodocea serrulata*: Viana et al., 2020; *E. acoroides*: Artika et al., 2020; Artika et al., 2021; *Zostera capensis*: Beltrand et al., 2022). However, when the optimal temperature for a species is surpassed, heat stress reduces these two traits (*Posidonia oceanica*: Traboni et al., 2018; *Halophila ovalis*: Ontoria et al., 2020, *Halophila stipulacea*: Viana et al., 2020). Co-inhabiting species can have different thermal optima (Collier et al., 2011), and their trait responses can give fundamental information on how future warming will affect seagrass communities. Traits can therefore inform about the responses of seagrass to environmental change, these responses being species-specific or general among seagrass species. The diversification of research to different species and bioregions is therefore fundamental to predict how seagrasses will deal with future global change scenarios, as even co-inhabiting species may respond in different ways (Agawin et al., 2001).

The prevalence of the use of morphological traits (55.9%) among all other trait categories is worth mentioning (biochemical 31.5%; physiological 29.1%; growth 28.3%; mechanical 15.7%; reproductive 10.2%). This is likely explained by their relatively easy and inexpensive measurements compared to physiological measurements of e.g., photosynthetic efficiency (Hernán et al., 2016; Llagostera et al., 2016) or enzymatic activity (Alexandre et al., 2004; Alexandre et al., 2010), which require specialized equipment, technical staff and laboratories. There are, therefore, extrinsic economical and technical reasons that constrain scientific questions in seagrass research. This trait type imbalance may impede a deeper understanding of responses of seagrasses to abiotic drivers, as physiological and biochemical indicators are recommended over morphological ones for early stress detection in seagrasses (Roca et al., 2016).

The study of morphological (52%), biochemical (50%) and growth traits (34.8%) under the biotic filter was more balanced compared to the abiotic filter. Most of the traits were studied in response to eutrophication (36.9%) and/or herbivory (23.9%). Eutrophication is considered one of the main threats to seagrasses, as it can lead to a phase shift in primary producers from seagrass to macroalgal dominance (Duarte, 1995; Orth et al., 2006; Burkholder et al., 2007; Waycott et al., 2009). Eutrophication affects different compartments of the ecosystem (seagrass, micro- and macroalgae, epiphytic organisms), changing their relative abundances and causing changes in the light penetration or the redox potential in the sediment (Burkholder et al., 2007). Seagrass species respond to eutrophication through their traits, again highlighting their value as environmental change indicators. Under eutrophication, *Cymodocea nodosa* increases the nutrient content in its leaves, while reducing its fiber content and biomechanical properties (Jiménez-Ramos et al., 2018a). Similarly, *P. oceanica* increases the nutritional quality of its leaves under fertilization, while reducing plant growth (Ravaglioli et al., 2018). In the case of herbivory, seagrass plants respond to this driver using a comprehensive array of traits, including growth compensation, changes in their nitrogen content or mobilization of carbohydrates (Sanmartí et al., 2014) and their morphology and growth form can predict grazing impacts on a global scale (Poore et al., 2012).

One of the main questions posed in the conceptual TBF is the relative importance of the abiotic *vs* the biotic filters (**Figure 1**: Hypothesis 2) to better understand the fundamental and realized niches of seagrass species. The available trait-based studies have focused separately on the fundamental and realized niches of seagrass species. We found that only 17.1% of the studies included both biotic and abiotic factors simultaneously, which indicates the existence of a knowledge gap in this topic. Trait-based approaches suggest the study of the convergence and divergence of trait distributions to unravel the relative effects of the abiotic and biotic filters (see review by Grime, 2006). Herbaceous plant communities tend to diverge at the species level but converge at the trait level (Fukami et al., 2005), indicating that environmental forces select for functional groups but not for species identities, which are historically contingent. This finding exemplifies the two schools of thought in ecological community assembly. On one hand, it has been suggested that different species coexist, occupying different niches (Diamond, 1975). On the other hand, members of the same plant community tend to exhibit similarity in plant traits, therefore showing overlapping niches (Clements, 1916).

The drivers that shape communities, namely environmental drivers, competition, and disturbance regime, can act at different spatial scales (Díaz et al., 1998; Pierce et al., 2007). To disentangle these effects, it is necessary to calculate the functional diversity (FD) and, specifically, functional trait dissimilarity among species within and among communities (Petchey and Gaston, 2002; De Bello et al., 2009). If the functional dissimilarity is lower than a set of random species (null model, see Götzenberger et al., 2016), this indicates trait convergence due to environmental filtering, whereas the opposite indicates trait divergence, and therefore a dominance of competition and/or a disturbance regime allowing for differential life-history strategies (Mason et al., 2007; Mouillot et al., 2007; Petchey et al., 2007). Despite the knowledge that seagrass traits change under environmental drivers (Roca et al., 2016) and that these traits affect interspecific competition (Moreira-Saporiti et al., 2021b), there are only a handful of examples in which trait convergence and divergence (**Figure 1**: Hypothesis #2) have been tested, and uniquely in *Z. marina* at the intraspecific level. *Z. marina* communities have a higher trait diversity with higher genetic relatedness among genotypes, indicating that trait divergence may be selected among competing genotypes (Stachowicz et al., 2013). Similarly, niche differentiation through trait divergence is suggested as an explanation for the positive correlation between its genotypic and trait diversity (Abbott et al., 2017). However, transplant experiments have shown that the species morphology changes under local environmental conditions, resembling the morphology of local populations (Ruesink, 2018). The results of these studies indicate that divergence occurs at the local level among competing plants, while convergence seems to occur at a larger scale.

The large body of literature on response traits under abiotic and biotic factors shows that there is a wealth of data that can be reassessed to answer questions in the context of the TBF presented here. In addition, the study of response traits uses a variety of nomenclature that could not be included in this review (e.g. indicators, responses…), further increasing the volume of data available. Despite the presentation of neutral and niche assembly theories separately, both are not incompatible. While some species can be stochastically eliminated from the local community (not reaching a suitable habitat or a random event leading to local extinction, see Sp6 in **Figure 1**), the subset of species that successfully colonized a community undergo a process of niche assembly. The disentanglement of fundamental and realized niches under a TBF is currently unexplored in seagrasses, providing an opportunity to answer fundamental research questions under global change that includes both abiotic and biotic drivers.

### 3.4 Phylogenetic and genotypic control of seagrass traits

The study of the phylogenetic and genotypic control of traits is quite prevalent in the seagrass literature (23.3% of studies, **Figure 3**). There is ample evidence that genotypic richness covaries with phenotypic variation in functionally relevant traits, such as leaf morphology and shoot productivity within *P. australis* (Evans et al., 2016). In contrast, genetic diversity is a poor proxy for trait differentiation in *Z. marina* (Abbott et al., 2018). In *P. oceanica* a reproductive trait like flower abundance was negatively correlated to genotypic diversity and positively correlated to heterozygosity (Jahnke et al., 2015a), while there was a correlation of genetic indices and their response to environmental conditions (Jahnke et al., 2015b). Ecosystem functions like the accumulation of biomass and susceptibility to herbivory are also genotypically controlled in *Z. marina* (Tomas et al., 2011), with genotypes differing in key traits related to these processes. Similarly, nutrient uptake rates differed among genotypes of *Z. marina* (Hughes et al., 2009).

These studies confirm that there is genetic control of seagrass traits (**Figure 1**: Hypothesis #3) and, consequently, of ecosystem functions and services (Díaz et al., 2013). However, this control is species-specific. Knowledge gaps in this area lay in the lack of information from most of the seagrass species, as the genus *Zostera* and *Posidonia* accumulate 82.2% of the studies.

### 3.5 Intraspecific trait variability in seagrasses is key to their survival

In seagrass ecosystems, characterized by low plant species richness, intraspecific variation is likely to play a more important role than in terrestrial ecosystems. In comparison to terrestrial plant lineages, the taxonomic diversity of seagrass is low with all species belonging to four Alismatales families. Indeed, many temperate meadows are monospecific, and most tropical meadows consist of only a handful of co-occurring species (Short et al., 2007).

Species and populations can differ for the level of plasticity (i.e., amplitude of the genotypes’ reaction norm), which is a fundamental trait affecting genotype persistence in changing environments (Pazzaglia et al., 2021). The plasticity of populations and genotypes is given by different levels of genetic variability, encompassing clonal somatic mutations and epigenetic changes. Several studies have indicated that the intraspecific trait variability of seagrass species is key for their survival. For example, the plant size of *Z. marina* (the predominant species in the northern hemisphere) spans more than two orders of magnitude across its distribution range (Ruesink, 2018), and different genotypes show large differences in nutrient uptake capacity and key photosynthetic parameters when grown in a “common garden” (Hughes et al., 2009). Even putatively less plastic species such as *P. oceanica* display a large variation in the acclimation to environmental factors (e.g. heat, Marín-Guirao et al., 2018). This feature, potentially supported by high intra-specific and intra-clonal (epi-)genetic diversity, enables seagrasses to cope with major environmental changes (Maxwell et al., 2014) and has most likely contributed to their successful colonization of shallow coastal zones along five continents, despite their low taxonomic diversity. Intraspecific variability in traits does not only occur at the species level, but also at the shoot, rhizome and clone levels. Epigenetic differences are even present within the same rhizome, which foster clonal persistence both within the same shoot (Ruocco et al., 2021) and within the same leaf (Ruocco et al., 2019a; Ruocco et al., 2019b). Recent evidence even points out that within single clones, somatic mutations lead to differentiation of ramets (= clone mates), with the potential to result in phenotypic differences within clones (Yu et al., 2020).

This body of literature highlights the importance of intraspecific trait variability in the response of seagrasses to disturbances, their resilience and capacity for ecosystem functions provision. However, we found only one example of the simultaneous study of intra- and interspecific variability of structural and nutritional traits, which drive palatability and herbivory in seagrasses (Jiménez-Ramos et al., 2018b). Future research assessing the relative importance of inter- vs intraspecific variability in both response and effect traits (**Figure 1**: Hypothesis #4) will be necessary to understand the relative role of intra- and interspecific diversity in seagrass ecosystem functions.

### 3.6 Effect traits and seagrass ecosystem functions: Understanding complementarity, dominance, and environmental control of ecosystem functions

The links between effect traits and ecosystem functions were tested in 26.9% of the studies (**Figure 3**). Herbivory (38.4%) and primary production (23%) were the most studied functions. Morphological (53.8%) and biochemical (46.1%) traits were the most used in the assessment of functions (**Figure 3**). Examples include wave attenuation, which is explained by a combination of morphological and mechanical traits including blade stiffness, shoot density and leaf length (Bouma et al., 2005; Paul et al., 2012) or herbivory of *Z. noltei*, which is mediated by both structural and nutritional leaf traits (Martínez-Crego et al., 2016). Examples of more nuanced, indirect interactions between traits and functions include the reduction of the canopy height in *P. oceanica* by grazing, thereby increasing the predation risk on associated sea urchins (Pagès et al., 2012).

There are, therefore, clear mechanistic links between seagrass effect traits and ecosystem functions. However, at the community level, there is the question of whether effect traits control ecosystem functions through dominance (CWM) or complementarity (FD) (**Figure 1**: Hypothesis #5). In addition, the link between traits and ecosystem functions can be environmentally constrained (**Figure 1**: Hypothesis #6). A great number of studies on ecosystem functions included environmental metrics (71.1%).

The hypothesis of the control of ecosystem functions by functional complementarity (FD) versus dominance (CWM) have been barely tested in seagrass ecosystems, with only a handful of examples found in the literature review (**Table 2**). Regarding the dominance hypothesis, CWM has been found as a reliable predictor of primary production in marine and brackish plant communities, including *Z. marina* (Gustafsson and Norkko, 2019). Particularly, plant height had positive effects on primary production, while the effects of other traits were environmentally constrained (**Table 2**). In the case of carbon storage, geophysical attributes seem to constrain any effect of seagrass traits (Belshe et al., 2018). Complementarity alone was tested in one study (Abbott et al., 2017, **Table 2**), showing that the Rao quadratic entropy index of trait diversity can predict invertebrate abundance. We found only two studies assessing simultaneously the effect of dominance and complementarity on ecosystem functions. In the case of habitat provision for fishes, trait complementarity had no effect, while the dominance of structurally more complex plants positively affected fish abundance (Jones et al., 2021). In the case of primary production, dominance of taller plants with bigger leaves positively affected production (Angove et al., 2020), while complementarity was discarded as a significant driver.

**Table 2:** Studies included in the literature review which test ecosystem function provision by seagrass communities through trait dominance, complementarity, and environmental constraints (**Figure 1**: Hypothesis #5 and 6).

Ecosystem function	Seagrass species	Effect trait(s)	Hypothesis tested			Conclusion	Reference
			Dominance	Complementarity	Environmental constraint		
Carbon storage	*T. ciliatum*, *C. serrulata*, *C. rotundata*, *T. hemprichii, S. isoetifolium, H. uninervis*, *H. ovalis*, *H. stipulacea*	Above- and belowground biomass, nitrogen content, shoot density	Yes	No	Geophysical attributes	**Environmentally constrained**. No trait dominance effects	Belshe et al. (2018)
Primary production	*Z. marina*, *R. cirrhosa*, other brackish plant species	Maximum vegetative height, specific leaf area, leaf and root nitrogen, leaf and root δ^15^N and δ^13^C, maximum root length	Yes	No	Exposure gradient	**Dominance effect and environmental constraints.** Vegetative height had a positive effect on primary production. Effects of root N and leaf δ^15^N were constrained by the exposure gradient.	Gustafsson and Norkko (2019)
	*Z. marina*, algae species, brackish plant species	Life habit (longevity, environmental position), morphology (growth form, size), tolerance (salinity and wave exposure tolerance) traits	Yes	No	No	**Dominance effect** of the three trait categories included.	Jänes et al. (2017)
	*Z. marina*, *R. cirrhosa*, other brackish plant species	Median height, leaf area, median maximum root length, specific root length, leaf nitrogen content, leaf δ^15^N and δ^13^C	Yes	Yes	No	**Dominance effect** of plant height and leaf area. Effect of functional richness due to presence of extreme trait values, not because of complementarity effect.	Angove et al. (2020)
Habitat provision for fishes	*T. ciliatum*, *C. serrulata*, *C. rotundata*, *T. hemprichii, S. isoetifolium, H. uninervis*, *H. ovalis*, *H. stipulacea*, *E. acoroides*	Meadow structure (shoot density, leaves per shoot, canopy height, leaf length, leaf width), seagrass cover	Yes	Yes	Depth	**Dominance effect** of meadow structural complexity. **Environmental effect** of depth. No complementarity effects.	Jones et al. (2021)
Habitat provision for invertebrate grazers	*Z. marina* (intraspecific study using different *Z. marina* genotypes)	17 traits, summarized: biomass accumulation, growth rate, morphology, nutrient uptake rate, leaf phenolic content, photosynthetic parameters	No	Yes	No	**Complementarity effect** (Rao quadratic entropy) of trait diversity on invertebrate grazer abundance.	Abbott et al. (2017)

There were, however, several studies on *Z. marina* assessing both the dominance and complementarity effects of genotypic diversity on ecosystem functions. Primary production is influenced by genotypic diversity of *Z. marina* at the plot level (Abbott et al., 2017). There is also evidence of intraspecific niche complementarity in the partitioned nutrient uptake of genotypes of *Z. marina* (Hughes et al., 2009). Dominance and complementarity hypotheses have been tested simultaneously in one study in *Z. marina* (Hughes and Stachowicz, 2011). Biomass production was higher in polycultures (i.e., higher complementarity) at high disturbance levels, whereas under no disturbance, monocultures (i.e., dominance) outperformed polycultures. Additionally, polycultures outperformed monocultures in shoot and biomass production under a macroalgal bloom. It is worth mentioning that, despite not being included in the literature review due to not being focused on the study of traits, there have been studies in communities including *Z. marina* linking taxonomic richness to resistance to shading (Gustafsson and Boström, 2013) and complementarity to increased biomass production (Salo et al., 2009).

The scarcity of trait complementarity *vs* dominance data on seagrasses highlights the complexity of assessing their relative importance, particularly under a changing environment. To test the dominance and complementarity hypotheses it is fundamental to find effect traits with proven mechanistic relationships with ecosystem functions. These relationships may be environmentally controlled and therefore it is necessary to include relevant environmental metrics in the study of ecosystem functions (van der Plas et al., 2020). This has been barely tested in seagrass communities and only in the case of three ecosystem functions (primary production, habitat provision for invertebrates and fishes and carbon storage, see **Table 2**). There is therefore a big knowledge gap in our understanding of how the functional traits of seagrass communities are linked to ecosystem functions, and how this provision will be altered under global change.

### 3.7 Vulnerability of seagrass ecosystem function and service provision under global change

The worldwide rate of seagrass loss and the numerous threats to seagrass ecosystems (Orth et al., 2006; Waycott et al., 2009) call for the assessment of the vulnerability of the ecosystem functions provided by seagrasses. Therefore, it is necessary to study the correlation between response and effect in seagrass ecosystems (**Figure 1**: Hypothesis #7).

As stated in previous sections, the study of the response of traits to environmental drivers is common, particularly in the case of temperature or light (Tanaka and Nakaoka, 2006; Mota et al., 2018). Traits are sensitive indicators of plant stress under environmental change (Roca et al., 2016). When it comes to ecosystem functions, their vulnerability is generally discussed in terms of seagrass loss, i.e., the loss of the seagrass meadows would mean the end of certain ecosystem function provision (Trevathan-Tackett et al., 2018). However, one important missing link is the identification of response traits that drive function effects. The rationale is that, despite the presence of seagrasses, a change in their trait values or the replacement by another species or taxa with different traits may alter ecosystem function provision. This hypothesis has not been explicitly tested in seagrass ecosystems (**Figure 1**: Hypothesis #7).

The concept of ecosystem service has gained increasing importance in the last two decades, as a tool to couple science with environmental policymaking and management (Costanza et al., 1997; Costanza et al., 2017). The identification of effect traits responsible for the provision of ecosystem services is of fundamental importance to develop a taxon-independent metric that could be incorporated into policymaking and guide coastal management strategies. Ruiz-Frau et al. (2017) classified ecosystem functions performed by seagrass in ecosystem services based on the TEEB - The Economics of Ecosystems and Biodiversity categorization created in TEEB (2009). For example, fisheries are classified as food provision, while carbon burial and storage are classified as gas and climate regulation. Knowing which functions underlie each ecosystem service, and how to relate simple trait metrics to ecosystem function and service vulnerability, is fundamental to achieve a holistic view of seagrass response, function provision and service provision under a changing environment.

## 4 Conclusion

TBFs (trait-based response-effect frameworks) are a powerful tool to address ecological questions in all fields of study, both terrestrial and marine. The synthesis of a comprehensive TBF based on previous knowledge allowed for a holistic view of traits, from their response to environmental drivers to ecosystem service provision.

The proposal to apply a TBF to seagrass ecology acknowledges the importance of considering the scientific advances of other research fields in order to push marine research forward. The application of a TBF to seagrasses appears as a powerful avenue to unveil new insights on the functioning of these important ecosystems, particularly in face of their special evolutionary history and narrow phylogenetic origin. We revealed that there is a wealth of data on seagrass response and effect traits, and on seagrass ecosystem functions, which allow a great potential to re-analyze existing data under a TBF perspective so that new research questions and hypotheses may be tested. In addition, there is a variety of nomenclature to refer to traits in seagrass research, further increasing the volume of data that could be reassessed under a TBF perspective but was not included in this review.

Most of the hypotheses of the TBF have not yet been formally tested. There is much evidence that stochastic processes (Hypothesis #1) have a lower relative importance than niche-based processes, both in the dispersal and vegetative stages of community assembly (Hypothesis #2). Additionally, traits are under a certain level of genotypic control (Hypothesis #3), but this could be highly trait dependent. Intraspecific diversity seems to be one of the mechanisms by which seagrasses respond to environmental drivers, and its understanding will prove fundamental to predict the response of seagrass to global change (Hypothesis #4). Ecosystem function provision by seagrass communities is generally controlled by trait dominance, but genotypic complementarity has also been shown to affect ecosystem functions, showing the need to understand the link between genotypic and functional trait diversity (Hypothesis #5). Additionally, only a handful of functions have been studied and the importance of dominance or complementarity can be environmentally constrained, as it is the case for primary production and carbon storage (Hypothesis #6).

Despite the positive signs of seagrass recovery in Europe and the United States, we cannot ignore the fact that the world is experiencing fast and unprecedented changes. The use of a TBF that assesses the vulnerability of ecosystem function and service provision (Hypothesis #7) can help to understand which ecosystem services may be compromised by the changes in species traits or species abundances. Therefore, the translation of biological and ecological seagrass research into a framework explicitly considering ecosystem services will prove fundamental for the development of comprehensive policies and for the informed management of seagrass ecosystems. However, mechanistic links between traits, functions and services will have to be resolved, further indicating the need for the mechanistic understanding of the traits that underpin ecosystem functions and services.

In an era in which global open data storage and sharing is becoming a central part of research, there is real need for a seagrass trait database, which has been developed at the Centro de Ciências do Mar (CCMAR, Portugal) in collaboration with the Portuguese national bioinformatics research infrastructure (http://biodata.pt/Elixir.pt). The Seagrass TraitDB (https://bio.tools/seagrasstraitdb) adopts standardized file formats, metadata, vocabularies, and identifiers so that it is compatible with global plant trait databases such as TRY (Kattge et al., 2020). It validates, stores, and disseminates MIAPPE-compliant data (https://www.miappe.org) and uses plant trait ontology to describe phenotypic traits of seagrasses. This tool will prove fundamental for the development of holistic and global research on seagrasses and a great opportunity for the application of the proposed TBF. We urge seagrass scientists to contribute to this data base.

We believe that the adoption of the concepts presented in this manuscript in seagrass research will aid the assessment of ecosystem services provision, improving the awareness of humankind on the importance of seagrass meadows worldwide.

## Data availability statement

The original contributions presented in the study are included in the article/**Supplementary Material**. Further inquiries can be directed to the corresponding author.

## Author contributions

This review and conceptual TBF was initiated as part of the Euromarine workshop “TRAITGRASS” led by GP and RS. All the authors contributed to the initial discussion that led to the production of this manuscript. AM-S did the systematic review for the manuscript. AM-S wrote the initial draft with significant contributions from all the authors. All the authors critically revised the different versions of the manuscript. All authors contributed to the article and approved the submitted version.
