# Supplementary material for: A trait-based framework for seagrass ecology: Trends and prospects
Source: Front Plant Sci. 2023 Mar 20;14:1088643. doi: 10.3389/fpls.2023.1088643 (PMC10067889; doi:10.3389/fpls.2023.1088643)
Supplement: Supplementary file 1 [file DataSheet_1.docx]

**SUPPLEMENTARY MATERIAL 1**

Metadata of the systematic review process following the ROSES protocol (Haddaway et al. 2018).

| **Protocol** | | ROSES  Haddaway et al. (2018)  https://link.springer.com/article/10.1186/s13750-018-0121-7 |
| --- | --- | --- |
| **Title** | Title | A trait-based framework to seagrass ecology: trends and prospects |
| **Type of review** | Type of review | Systematic review |
| **Authors contacts** | Author contacts | Agustín Moreira Saporiti (am.saporiti@gmail.com) |
| **Abstract** | Structured summary | See abstract in manuscript |
| **Background** | Background | See manuscript |
| **Objective of the review** | | Screen methods and concepts used in the study of seagrass traits, with a specific focus on trait-based approaches and functional ecology. |
| **Methods** | | |
| **Searches** | Search strategy | Web-search |
|  | Search string | “Seagrass”+”trait” and “*seagrass species*”+”trait” |
|  | Languages | English |
|  | Languages-grey literature | No grey literature included. |
|  | Web-based search engines | Google Scholar |
|  | Estimating the comprehensiveness of the search | All articles published subjected to the inclusion criteria. |
| **Article screening and study inclusion criteria** | Screening strategy | Screening of title, abstract and keywords |
|  | Inclusion criteria | “trait” has to be present in title, abstract and/or keywords in reference to seagrass |
|  | Reasons for exclusion | “trait” is used for the study of seagrass associated fauna, epiphytes, macroalgae or environment. |
| **Screening process** | Number of search results from web-based search engines. | 21100 |
|  | Number of articles retained following title and abstract screening. | 380 |
|  | Number of articles retained after applying exclusion criteria | 243 |
|  | Number of articles retained after removing duplicates | 231 |
|  | Number of articles retained after removing misclassified articles | 212 |
|  | Number of articles retained after removing gray literature | 193 |
|  | Final number of articles included in the review process | 193 |
| **Data extraction** | No data extraction | |
| **Data synthesis and presentation** | Type of synthesis | Narrative and quantitative |
|  | Narrative synthesis strategy | Summary of trait-based and functional ecology concepts used in seagrass research |
|  | Quantitative synthesis strategy | Quantification of study types, traits types, environmental drivers and functions studied in seagrass, both with and without trait-based approaches. |
|  | Assessment of risk of publication bias | The inclusion of articles that include the word “trait” ignores a lot of publications in which traits are being studied, but the word “trait” is not used. However this compromise was necessary to target trait-based studies. |

**SUPPLEMENTARY MATERIAL 2**

Complete database used for this manuscript. Excel file: Database_Review.xlsx

**SUPPLEMENTARY MATERIAL 3: STUDIES CLASSIFIED BY YEAR, BIOREGION AND SPECIES**

| **Table 1.** Number of studies that included the word “trait” and, within them, classified as “TBF”, categorized a) per year, b) per bioregion (according to Short et al. 2007) and c) per species. | | |  |
| --- | --- | --- | --- |
| a) Per year | Trait studies | TBF | |
| 1988 | 1 | 0 | |
| 1989 | 0 | 0 | |
| 1990 | 0 | 0 | |
| 1991 | 0 | 0 | |
| 1992 | 0 | 0 | |
| 1993 | 0 | 0 | |
| 1994 | 0 | 0 | |
| 1995 | 0 | 0 | |
| 1996 | 1 | 0 | |
| 1997 | 0 | 0 | |
| 1998 | 1 | 0 | |
| 1999 | 1 | 0 | |
| 2000 | 2 | 0 | |
| 2001 | 2 | 0 | |
| 2002 | 1 | 0 | |
| 2003 | 3 | 0 | |
| 2004 | 2 | 0 | |
| 2005 | 2 | 0 | |
| 2006 | 3 | 0 | |
| 2007 | 7 | 0 | |
| 2008 | 4 | 0 | |
| 2009 | 4 | 0 | |
| 2010 | 2 | 0 | |
| 2011 | 8 | 0 | |
| 2012 | 11 | 1 | |
| 2013 | 7 | 0 | |
| 2014 | 13 | 1 | |
| 2015 | 5 | 0 | |
| 2016 | 14 | 2 | |
| 2017 | 12 | 3 | |
| 2018 | 23 | 3 | |
| 2019 | 9 | 3 | |
| 2020 | 28 | 10 | |
| 2021 | 22 | 4 | |
| 2022 | 5 | 2 | |
| b) Per bioregion | Trait studies | TBF | |
| Temperate North Atlantic | 45 | 7 | |
| Tropical Atlantic | 24 | 5 | |
| Mediterranean | 71 | 7 | |
| Temperate North Pacific | 46 | 10 | |
| Tropical Indo-Pacific | 46 | 7 | |
| Temperate Southern Oceans | 30 | 5 | |
| c) Per species | Trait studies | TBF | |
| *Amphibolis antarctica* | 8 | 0 | |
| *Amphibolis girffithii* | 6 | 0 | |
| *Cymodocea nodosa* | 23 | 1 | |
| *Cymodocea rotundata* | 15 | 3 | |
| *Cymodocea serrulata* | 16 | 4 | |
| *Enhalus acoroides* | 13 | 3 | |
| *Halodule pinifolia* | 3 | 0 | |
| *Halodule uninervis* | 12 | 3 | |
| *Halodule wrightii* | 10 | 1 | |
| *Halophila baillonis* | 3 | 0 | |
| *Halophila beccarii* | 5 | 0 | |
| *Halophila capricorni* | 1 | 0 | |
| *Halophila decipiens* | 8 | 0 | |
| *Halophila engelmannii* | 2 | 0 | |
| *Halophila gaudichaudii* | 1 | 0 | |
| *Halophila nipponica* | 1 | 0 | |
| *Halophila ovalis* | 19 | 5 | |
| *Halophila spinulosa* | 2 | 0 | |
| *Halophila stipulacea* | 12 | 3 | |
| *Halophila sulawesii* | 1 | 0 | |
| *Halophila tricostata* | 2 | 0 | |
| *Heterozostera polychlamys* | 1 | 0 | |
| *Phyllospadix iwatensis* | 5 | 0 | |
| *Phyllospadix japonicus* | 2 | 0 | |
| *Phyllospadix serrulatus* | 2 | 0 | |
| *Phyllospadix torreyi* | 3 | 0 | |
| *Posidonia angustifolia* | 2 | 0 | |
| *Posidonia australis* | 11 | 1 | |
| *Posidonia coriacea* | 6 | 0 | |
| *Posidonia denhartogii* | 3 | 0 | |
| *Posidonia kirkmanii* | 2 | 0 | |
| *Posidonia oceanica* | 36 | 3 | |
| *Posidonia ostenfeldii* | 4 | 0 | |
| *Posidonia robertsoniae* | 2 | 0 | |
| *Posidonia sinuosa* | 6 | 0 | |
| *Ruppia cirrhosa* | 3 | 2 | |
| *Ruppia maritima* | 10 | 1 | |
| *Ruppia megacarpa* | 4 | 0 | |
| *Ruppia tuberosa* | 1 | 0 | |
| *Syringodium filiforme* | 5 | 1 | |
| *Syringodium isoetifolium* | 13 | 3 | |
| *Thalassia hemprichii* | 19 | 4 | |
| *Thalassia testudinum* | 12 | 3 | |
| *Thalassodendron ciliatum* | 6 | 3 | |
| *Thalassodendron pachyrhizum* | 5 | 0 | |
| *Zostera asiatica* | 2 | 0 | |
| *Zostera caespitosa* | 4 | 0 | |
| *Zostera capensis* | 2 | 1 | |
| *Zostera caulescens* | 3 | 0 | |
| *Zostera japonica* | 6 | 1 | |
| *Zostera marina* | 58 | 13 | |
| *Zostera muelleri* | 12 | 0 | |
| *Zostera nigricaulis* | 5 | 0 | |
| *Zostera noltii* | 21 | 0 | |
| *Zostera novazelandica* | 2 | 0 | |
| *Zostera tasmanica* | 3 | 0 | |

**SUPPLEMENTARY MATERIAL 4: CATEGORIES DEFINITION**

| **Table 1.** Definition of the categories in which the studies have been classified. | |
| --- | --- |
| **Category** | **Definition and criteria** |
| **Trait category** | |
| Biochemical | Study of the biochemical composition of seagrass e.g. nitrogen, carbohydrates, fatty acids, secondary metabolites… |
| Growth | Study of growth of seagrass e.g. shoot production, leaf growth, rhizome elongation… |
| Mechanical | Study of sturdiness or robustness of seagrass e.g. flexibility, structural carbon content, force-to-break… |
| Morphological | Study of any morphological characteristic of seagrass e.g. leaf area, rhizome diameter, root length… |
| Physiological | Study of physiological processes e.g. photosynthetic parameters. |
| Reproductive | Study of reproductive structures of seagrass e.g. number of flowers, seed production… |
| **Study type** | |
| Dispersal | Study of dispersal mechanisms of seagrass |
| Abiotic filter | Study of the response traits of seagrass under abiotic environmental drivers |
| Biotic filter | Study of response traits of seagrass under biotic interaction, among seagrass species or between seagrass and other compartments of the ecosystem (herbivores, epiphytes, macroalgae…) |
| Function | Study of the provision of ecosystem functions and processes by seagrass related to seagrass traits. |
| Phylogeny | Study of seagrass genotypes, genes and gene expression in connection to seagrass traits. |
| **Dispersal** | |
| Seed/  Propagule establishment | Seed and propagule establishment in relation to their traits. Ecosystem process directly linked to dispersal. |
| Seed/Vegetative dispersal | Seed and vegetative fragments dispersal linked to their traits. |
| **Abiotic filter** | |
| Light/Turbidity | Light availability, penetration or shading. |
| Nutrients | Nutrients concentration, availability or enrichment in the water column and/or sediments |
| Temperature | Seawater temperature |
| Salinity | Seawater salinity |
| CO2/pH | CO2 enrichment, water acidification and/or alcalinity |
| Substrate | Type of substrate where seagrass grows or settles, e.g. sand, mud, rock |
| Grain size | Grain size distribution of seagrass substrate |
| Sediment burial/loading | Sediment addition and resupension on or within seagrass meadows |
| Depth | Effects of depth on seagrass |
| Desiccation/Air exposure | Intensity or length of seagrass desiccation. Included effects of tidal elevation. |
| Hydrodynamics | Effects of hydrodynamic regime, wave energy, currents and meadow exposure |
| Site | Site specific effects linked to other environmental drivers, which are not necessarily quantified. |
| Physical removal/Dredging | Effects of physical removal of seagrass by dredging, mooring or other phenomenons |
| Latitude/Climate | Latitude, climatic conditions or other large scale processes |
| Disturbance regime | Frequency of disturbance, which can be linked to other environmental drivers which are not necessarily quantified |
| Season | Season specific conditions |
| Heavy metal pollution | Concentration of heavy metals in seawater, e.g. chromium, copper… |
| Aquaculture/Other anthropogenic impacts | Effects of aquaculture practices and other anthropogenic impacts which can be linked to other environmental drivers, which are nor necessarily quantified |
| Estuary condition/gradient | General estuarine conditions, generally linked to other environmental drivers that are not necessarily quantified |
| Time/Temporal study | Temporal study, with repeated measures along a certain time scale. |
| Weather conditions | Weather conditions effects on seagrass, e.g. precipitation, cloud coverage |
| Freshwater input | Coastal input of freshwater on seagrass meadows |
| Other abiotic factors | Included abiotic factors that only appeared once in the database. List: collection method, O2 concentration, spatial scale, ocean upwelling, volcanic explosion, light cycle, siltation, time/temporal scale |
| **Biotic filter** | |
| Herbivory | Effects of herbivores on seagrass traits |
| Epiphytic growth/  abundance | Effects of epiphytic organisms growth and abundance on seagrass |
| Macroalgae abundance | Effects of macroalgae abundance on seagrass |
| Organic matter | Organic matter concentration in the water column and/or sediments |
| Microbial community/  processes | Interactions or effects of the microbial community or microbial processes on seagrass |
| Coral reef/mangrove presence | Effect or interaction of seagrasses with coral reefs and mangroves |
| Eutrophication | Effect of eutrophication and eutrophication related conditions on seagrasses. Linked to macroalgae abundance, epiphytic abundance and nutrients. |
| Interspecific competition/Plant interactions | Interactions and processes among seagrass plants related to light and nutrient competition. Linked to the biotic filter, but formally defined as an ecosystem process. |
| **Functions and processes** | |
| Carbon storage/  Sequestration | Carbon storage in the sediment, and carbon sequestration processes by seagrasses |
| Flow and wave attenuation | Flow attenuation due to the seagrass canopy. |
| Habitat provision | Habitat provision for fauna. |
| Herbivory (Function) | Food provision to herbivores. |
| Litter production/  decomposition | Production and decay of seagrass litter. |
| Nutrient uptake/Filtering | Uptake of inorganic nutrients by seagrass. |
| Primary production | Seagrass primary production. |
| Sediment trapping/  Resuspension prevention | Trapping of sediment particles. |
| Interspecific competition/Plant interactions | Interactions and processes among seagrass plants related to light and nutrient competition. Linked to the biotic filter, but formally defined as an ecosystem process. |
| Predation | Predation of associated fauna within seagrass meadows. |
| Seagrass mutualisms | Mutualism of seagrass with other organisms, e.g. lucinid clams |
| Exchange/fluxes of gas and nutrients | Exchange of nutrients and gases of seagrass meadows with other compartments of the ecosystem. |
| Nitrogen fixation | Nitrogen fixation within seagrass ecosystems. |
| **Phylogeny** | |
| Genotype/Genes | Studies of the connection between genotype and seagrass traits |
| Phenotype | Studies of the seagrass phenotypes, phenotype plasticity and their influence on seagrass traits |

**SUPPLEMENTARY MATERIAL 5: STUDIES CLASSIFICATION**

| **Table 1.** Classification of studies including the word “trait” and TBF studies in study types, subcategories and trait categories. | | | | | | |
| --- | --- | --- | --- | --- | --- | --- |
|  | | | | | **Trait studies** | **TBF studies** |
| **Total** | | | | | 193 | 29 |
| **Dispersion** | | | | | 7 | 0 |
|  | **Subcategories** | | | |  |  |
|  |  | | Seed/Propagule dispersion | | 5 | 0 |
|  |  | | Seed/Propagule settlement | | 3 | 0 |
|  | **Trait categories** | | | |  |  |
|  |  | | Morphological | | 4 | 0 |
|  |  | | Biochemical | | 1 | 0 |
|  |  | | Reproductive | | 1 | 0 |
|  |  | | Mechanical | | 3 | 0 |
|  |  | | Growth | | 2 | 0 |
|  |  | | Physiological | | 1 | 0 |
| **Dispersal: Abiotic and biotic filters connections** | | | | |  |  |
| **Abiotic filter** | | | | | 127 | 18 |
|  | **Subcategories** | | | |  |  |
|  |  | | Light/Turbidity | | 22 | 0 |
|  |  | | Nutrients | | 31 | 3 |
|  |  | | Temperature | | 36 | 8 |
|  |  | | Salinity | | 7 | 1 |
|  |  | | CO2/pH | | 8 | 1 |
|  |  | | Substrate | | 5 | 0 |
|  |  | | Grain size | | 2 | 1 |
|  |  | | Sediment burial/loading | | 8 | 1 |
|  |  | | Depth | | 14 | 1 |
|  |  | | Desiccation/Air exposure | | 8 | 0 |
|  |  | | Hydrodynamics | | 16 | 2 |
|  |  | | Site | | 8 | 0 |
|  |  | | Physical removal/Dredging | | 5 | 0 |
|  |  | | Latitude/Climate | | 9 | 0 |
|  |  | | Disturbance regime | | 4 | 0 |
|  |  | | Season | | 12 | 1 |
|  |  | | Heavy metal pollution | | 3 | 0 |
|  |  | | Aquaculture/Other anthropogenic impacts | | 4 | 1 |
|  |  | | Estuary condition/gradient | | 2 | 0 |
|  |  | | Weather conditions | | 3 | 1 |
|  |  | | Freshwater input | | 2 | 1 |
|  |  | | Other abiotic factors | | 7 | 1 |
|  | **Trait categories** | | | |  |  |
|  |  | | Morphological | | 71 | 12 |
|  |  | | Biochemical | | 40 | 5 |
|  |  | | Reproductive | | 13 | 0 |
|  |  | | Mechanical | | 20 | 1 |
|  |  | | Growth | | 36 | 8 |
|  |  | | Physiological | | 37 | 6 |
| **Biotic filter** | | | | | 46 | 9 |
|  | **Subcategories** | | |  |  |  |
|  |  | Herbivory | | | 11 | 3 |
|  |  | Epiphytic growth/abundance | | | 2 | 1 |
|  |  | Macroalgae abundance | | | 4 | 0 |
|  |  | Organic matter | | | 6 | 0 |
|  |  | Microbial community/processes | | | 2 | 0 |
|  |  | Coral reef/mangrove presence | | | 2 | 1 |
|  |  | Eutrophication | | | 17 | 3 |
|  |  | Interspecific competition/Plant interactions | | | 7 | 3 |
|  |  | Animal vector | | | 1 | 0 |
|  |  | Leaf position within shoot | | | 1 | 0 |
|  | **Trait categories** | | | |  |  |
|  |  | Morphological | | | 24 | 6 |
|  |  | Biochemical | | | 23 | 3 |
|  |  | Reproductive | | | 3 | 0 |
|  |  | Mechanical | | | 9 | 0 |
|  |  | Growth | | | 16 | 3 |
|  |  | Physiological | | | 10 | 2 |
| **Abiotic:Biotic connections** | | | | | 33 | 4 |
| **Functions and processes** | | | | | 52 | 15 |
|  | **Subcategories** | | | |  |  |
|  |  | | Carbon storage/Sequestration | | 4 | 2 |
|  |  | | Flow and wave attenuation | | 6 | 0 |
|  |  | | Habitat provision | | 4 | 3 |
|  |  | | Herbivory (Function) | | 20 | 0 |
|  |  | | Litter production/decomposition | | 1 | 0 |
|  |  | | Nutrient uptake/Filtering/Fixation/Fluxes | | 6 | 2 |
|  |  | | Primary production | | 12 | 6 |
|  |  | | Sediment trapping/Resuspension prevention | | 1 | 0 |
|  |  | | Predation | | 1 | 1 |
|  |  | | Seagrass mutualisms | | 1 | 0 |
|  | **Trait categories** | | | |  |  |
|  |  | | Morphological | | 28 | 11 |
|  |  | | Biochemical | | 24 | 5 |
|  |  | | Reproductive | | 2 | 0 |
|  |  | | Mechanical | | 13 | 0 |
|  |  | | Growth | | 6 | 3 |
|  |  | | Physiological | | 5 | 2 |
| **Phylogeny** | | | | | 45 | 7 |
|  | **Subcategories** | | | |  |  |
|  |  | | Gene/Genotype | | 31 | 5 |
|  |  | | Phenotype | | 15 | 2 |
|  | **Trait categories** | | | |  |  |
|  |  | | Morphological | | 23 | 3 |
|  |  | | Biochemical | | 7 | 2 |
|  |  | | Reproductive | | 4 | 0 |
|  |  | | Mechanical | | 2 | 0 |
|  |  | | Growth | | 15 | 3 |
|  |  | | Physiological | | 11 | 3 |
| **Abiotic and Biotic Filters: Function connections** | | | | | 37 | 11 |
